# Comparative assessment of methicillin resistant *Staphylococcus aureus* diagnostic assays for use in resource-limited settings

**DOI:** 10.1186/s12866-019-1566-8

**Published:** 2019-08-22

**Authors:** A. Ayebare, L. M. Bebell, J. Bazira, S. Ttendo, V. Katawera, D. R. Bangsberg, M. J. Siedner, P. G. Firth, Y. Boum II

**Affiliations:** 1 Faculty of Medicine, Mbarara University of Science and Technology, Mbarara, Uganda; 2 Epicentre Mbarara Research Centre, Mbarara, Uganda; 3 Division of Infectious Diseases, Massachusetts General Hospital, Boston, MA USA; 4 Massachusetts General Hospital Center for Global Health, Boston, MA USA; 5 World Health Organization, Monrovia, Liberia; 6 Oregon Health & Science University-Portland State University School of Public Health, Portland, OR USA; 7 Massachusetts General Hospital Department of Anesthesia, Critical Care and Pain Medicine, Boston, MA USA

**Keywords:** Methicillin-resistant *Staphylococcus aureus*, MRSA, Carriage, Chromogenic agar, PCR, Africa

## Abstract

**Background:**

The rise of methicillin-resistant *Staphylococcus aureus* (MRSA) is a global health concern. Paucity of data on MRSA carriage prevalence and diagnostic methods in resource-limited settings hampers efforts to define the problem and plan an appropriate response. Additionally, high variability in cost and logistical characteristics of MRSA screening methods may impede infection control efforts. We compared the performance of locally-available chromogenic agar BD CHROMagar MRSA II and two PCR-based assays (Hain GenoQuick MRSA and Cepheid Xpert SA Complete) for the detection of asymptomatic MRSA carriage in nasal swabs.

**Results:**

During 2015, we enrolled 500 patients from five hospital wards at a Ugandan regional referral hospital. We found 30% prevalence of methicillin-sensitive *Staphylococcus aureus* (MSSA) nasal carriage, and 5.4% MRSA nasal carriage prevalence. Compared to a composite reference standard defined as a positive test result on any one of the three assays, Hain GenoQuick MRSA demonstrated the highest sensitivity (96%) followed by direct plating on CHROMagar at (70%), with the lowest sensitivity observed with Xpert SA Complete (52%). Cepheid Xpert provided the most rapid results (< 1 h) but was the most expensive (US $45–50/test). Substantially more labor was required for the Hain GenoQuick MRSA compared to Xpert SA Complete or CHROMagar tests.

**Conclusion:**

MRSA nasal carriage prevalence rates were low, and high diagnostic sensitivity was achieved using Hain GenoQuick MRSA. Chromogenic media had significantly lower sensitivity, but may represent a viable local option given its lower cost compared to PCR-based assays.

## Background

*Staphylococcus aureus* is one of the most common bacterial pathogens associated with both nosocomial and community-acquired infections [1]. Due to its commensal nature on human skin and mucous membranes, *S. aureus* spreads readily from person-to-person, causing infections in skin, soft tissues and the bloodstream [2]. The increasing use of antibiotics contributes to emergence of methicillin-resistant strains of *S. aureus* (MRSA) [3]. MRSA infections remain a significant cause of hospital-acquired infections [4, 5]; accounting for up to 75% of all *S. aureus* isolates from patients with skin and soft-tissue infections among patients seen in emergency departments in the United States [3].

Patients with invasive MRSA infections are often colonized with MRSA or acquire it from healthcare workers [6]. In many high-resource settings, patients are tested for MRSA carriage on hospital admission and may undergo isolation or decontamination to reduce MRSA transmission [7, 8]. Towards this end, several detection methods have been used to evaluate MRSA carriage, including polymerase chain reaction (PCR)-based assays and culture using chromogenic agar. Although there is no universal reference standard for MRSA testing, molecular methods such as PCR are often used in high-resource settings to detect nasal MRSA carriage, reducing reporting times from days to hours, compared to traditional culture techniques [9].

In resource-limited settings, however, few facilities use molecular methods due to high test costs, and lack of necessary equipment and appropriately-trained personnel. There is a paucity of data about the relative performance of laboratory tests to diagnose MRSA nasal carriage in resource-limited settings. Therefore, we assessed three screening methods for MRSA nasal carriage with varying characteristics of test speed, cost, human resource, laboratory requirements, and diagnostic sensitivity.

## Methods

### Participants, specimen and data collection, and quality control

We performed a cross-sectional study of 500 adult patients presenting for care to Mbarara Regional Referral Hospital (MRRH), a 608-bed hospital located in Mbarara, approximately 265 km southwest of Uganda’s capital Kampala. The patient population resides in a predominantly rural, agricultural area. Between January to October 2015, one hundred patients were enrolled consecutively from each of five hospital departments: inpatient surgery ward, in-patient medicine ward, in-patient maternity ward, general out-patient department, and the out-patient Immune Suppression Syndrome (ISS) Clinic, which exclusively serves patients living with HIV until the enrollment target was reached and then enrollment was begun in the next department. Adult participants ≥18 years of age cared for at any of the five hospital departments were eligible for enrollment. Exclusion criteria were age <  18 years, inability to speak English or the local language Runyankole, and inability for the participant or their next-of-kin to provide informed consent. Participants included were part of a clinical study assessing prevalence and correlates of MRSA and MSSA nasal carriage at a Ugandan regional referral hospital [10]. Study participants had their bilateral anterior nares swabbed with two sterile Dacron-tipped dual-swabs representing a combined sample of both nares. Each Dacron-tipped collection device has two swabs giving a total of four swabs. Three were used for the MRSA detection methods, and the remaining swab was kept refrigerated for retesting. Swabs were transported same-day to the Epicentre laboratory on-site for refrigeration and processing within 24 h. Quality control procedures were performed using commercially available bacteria strains ATCC 33591 MRSA and ATCC 25923 MSSA on each new lot of testing kits. All samples producing invalid and erroneous results were repeated once. Demographic characteristics were captured using investigator-designed questionnaires.

### BD CHROMagar MRSA II

One swab (Copan Tran-system Liquid Amies double swab) was inoculated directly onto nutrient-enriched selective agar media (CHROMagar MRSA II, BD Diagnostic Systems, Sparks, USA) and incubated under aerobic conditions at 37 °C for 24 h according to the manufacturer’s instructions. After 24 h, each plate was examined for colony growth and color. All mauve colonies underwent further identification including Gram stain and coagulase testing to be confirmed as MRSA. Culture-negative plates were further incubated and examined at 48 h and discarded if negative.

### Xpert SA nasal complete

The second swab was processed for the Cepheid Xpert SA Nasal Complete assay (Cepheid, Sunnyvale, USA) according to the manufacturer’s instructions and run on the Cepheid Xpert SA Complete platform. Fluorescent signals of target DNA, SA-*spa*/MRSA-*mecA* and *mecA* and SCC*mec*) were measured and results were provided automatically by the GeneXpert machine. Results were reported positive for *S. aureus* if the *spa* gene was detected above threshold limits, and samples were reported MRSA-positive if *spa*, *mecA* and SCC*mec* genes were all detected above threshold limits. The minimum cycle threshold (Ct) detection limit for all genes was a Ct of 10, and the maximum Ct detection limit for *spa*, *mecA* and SCC*mec* was 35, 36, and 38, respectively.

### Hain GenoQuick MRSA

The third swab was run on the Hain GenoQuick MRSA assay (Hain Life Science, Nehren, Germany) according to the manufacturer’s instructions. A Lack of MRSA-positive samples in the first 100 swabs and documented 30-fold differences in level of detection led study investigators to optimize the Hain GenoQuick MRSA amplification procedure [11]. The remaining 400 samples were tested under a revised protocol where double the amount of DNA lysate was used, improving the test’s level of detection. As a result of logistical shortage of Hain GenoQuick kits, only 70 of the initial 100 samples were retested using the revised protocol, and 470 total sample results are reported. Results were then interpreted as positive or negative for MRSA for the assay according to the manufacturer’s instructions.

### Data entry and analysis

Demographic data and microbiology results were manually entered into a REDCap database hosted at Partners Healthcare in Boston, USA [12]. Analyses were performed in Stata version 12 (StataCorp, College Station, USA). As our primary interest was in assessing test sensitivity as the most relevant measure for infection control purposes, we created a composite reference standard (CRS), defining a sample as positive by the CRS if it tested positive by one or more individual tests: 1) BD CHROMagar, 2) Cepheid Xpert SA Nasal Complete, 3) Hain GenoQuick. We then calculated sensitivity of each test compared to the CRS. We carried out activity/task analysis by observation of these three test methods to define outcomes, including cost, time, and logistical needs.

## Results

**Fig. 1:**
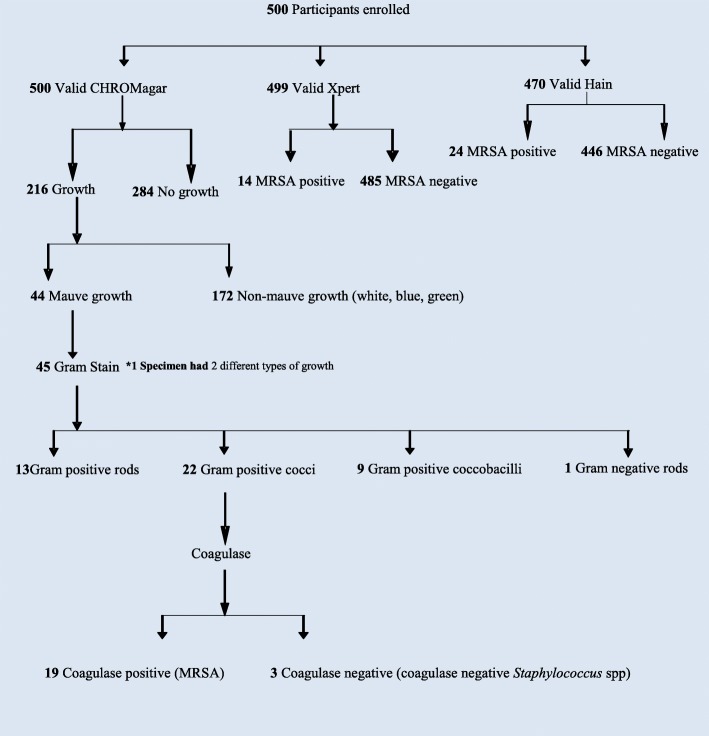
Flow diagram of samples collected and results from all three testing methods

We enrolled 500 participants, 100 from each of five hospital wards (Fig. 1). MRSA nasal carriage prevalence was 5.4% using the CRS (*n* = 27, 95% confidence interval [CI 3.6–7.8%]. Of 500 samples tested for MRSA using the Cepheid Xpert SA Complete assay, one yielded an invalid result, leaving 499 samples for analysis. Of these 499 samples, 14 were positive for MRSA (2.8%). Of 500 samples directly cultured onto CHROMagar MRSA II, 44 (8.8%) had characteristic mauve colony growth. Of these, 40 (90.9%) grew after 24 h and19 (43.2%) were confirmed MRSA with a positive tube coagulase test and Gram stain demonstrating gram-positive cocci. One of four plates with growth at 48 h (but not at 24 h) was also confirmed as MRSA. Of 470 samples tested for MRSA using the Hain GenoQuick MRSA revised protocol assay, 24 (5.1%) were MRSA positive.

The sensitivity of each assay for MRSA detection compared to the CRS was 96% (95% CI 81–100%) for Hain GenoQuick MRSA, 70% (95% CI 50–86%) for CHROMagar, and 52% (95% CI 32–71%) for GeneXpert SA nasal complete (Table 1). The cost per test was US $6.50–7.50 for CHROMagar MRSA with an 18–48 h turnaround time, $45–50 for GeneXpert SA Nasal Complete with a 1.25-h turnaround time, and $10–15 for Hain GenoQuick MRSA with a 3.5-h turnaround time (Table 2).

**Table 1:** Performance of each diagnostic test, compared to the composite reference standard (CRS), defined as a positive result on one or more of the individual tests: Hain GenoQuick, BD CHROMagar, Cepheid Xpert SA Nasal Complete

	^a^CRSMRSA (+) (*n* = 27)	CRS MRSA (−) (*n* = 472)	Sensitivity (%, 95 CI)
GenoQuick (*n* = 470)			
MRSA (+)	26	0	96 (81–100)
MRSA (−)	1	443	
CHROMagar (*n* = 500)			
MRSA (+)	19	0	70 (50–86)
MRSA (−)	8	472	
GeneXpert SA complete (*n* = 499)			
MRSA (+)	14	0	52 (32–71)
MRSA (−)	13	473	

*CI* Confidence interval

^a^A true positive culture was defined as MRSA detected by one or more of the three tests, and also called the composite reference standard (CRS)

**Table 2:** Comparison of detection methods by factors influencing decision to adopt a specific test

Variable	Assay		
	CHROMagar MRSA	Cepheid Xpert SA Complete	Hain GenoQuick
Initial investment cost (equipment, accessories)	US $14,400	US $43,000	US $23,900
Cost per test	US $6.50–7.50	US $45–50	US $10–15
Hands-on time per sample	4–5 min	< 1 min	~ 15 min
Number of Samples per Run	1 sample per plate	4 samples per run	12 samples per run
Testing time	18–48 h	< 1 h (50 mins)	2.5 h
Total turn-around-time	18–48 h	1.25 h	3.5 h
Human resource considerations	Technically demanding	Not technically demanding	Technically demanding
Challenges	Definite identification as MRSA requires confirming isolates as *S. aureus* with a coagulase test, which is not included in the test kit nor specified by the manufacturer	High failure rate –sample debris can clog pre-filter with particulates, leading to cartridge failure. No sample preparation is recommended by the manufacturer	Extraction, amplification and detection processes are cumbersome and hands-on. Assay needed to be optimized by doubling the amount of DNA extract, which was not specified by the manufacturer

## Discussion

The prevalence rate for MRSA nasal carriage was surprisingly low in our setting, and method comparison showed that the Hain GenoQuick MRSA assay demonstrated a high diagnostic sensitivity compared to the CRS. Chromogenic media had significantly lower sensitivity than the Hain GenoQuick MRSA.

Significant discrepant results between PCR assays may have resulted from, possibly due to MRSA genetic variants yielding undetectable or unstable amplification products [13–15]. Some studies have reported up to 30-fold higher level of detection for the Hain Geno Quick assay compared to the Cepheid Xpert SA Complete [11, 16, 17], and the relatively low sensitivity of the Cepheid Xpert SA Complete assay may have resulted from cycle time cut-off limits for the amplification target [15, 18, 19]. For the Hain GenoQuick MRSA assay, optimization was required to enhance detection, and required technical expertise and troubleshooting. Although precautions were taken to minimize PCR assay interference, it is also possible that invalid Cepheid GeneXpert results were due to the presence of PCR inhibitors in swab specimens, including mucin, blood, dust, or air bubbles. Compared to the CRS, culture-based methods demonstrated low sensitivity, which could be due to low bacterial density in nasal swab specimens. Similarly, participants’ current or recent antibiotic use may have rendered MRSA organisms non-viable on culture, or lowered the bacterial concentration below the PCR assays’ limits of detection. Compared to PCR-based assays, culture-based methods are disproportionately affected by bacterial concentrations, decreasing sensitivity [16, 17, 18].

The three methods differed greatly in cost, processing, turnaround time, and technical requirements with PCR platforms significantly more expensive especially due to up-front costs, some more required specialized equipment and considerable technical expertise but faster turnaround times may lead to downstream cost savings in infection prevention and control measures. The Hain GenoQuick MRSA assay is time-consuming, and requires technical expertise and specialized accessories/equipment, limiting its role in field-based MRSA screening whereas the Cepheid Xpert SA Complete assay is an easy-to-use and rapid point-of-care test requiring little hands-on time. Despite having the longest turnaround time and relatively low sensitivity culture-based method is easier to implement in resource-limited settings, balancing cost, technical expertise, and human resource requirements. Chromogenic methods are considered an improvement over conventional culture methods and surveillance testing of multiple body sites can significantly improve detection rates [20, 21].

Our study is one of the first to compare three method-variable assays for performance in a resource-limited setting. Strengths of our study include the large sample size, diverse patient population and use of multiple concurrent testing regimens. Weaknesses of the study include the need to optimize the Hain GenoQuick MRSA assay, and lack of molecular typing of MRSA grown in culture due to resource limitations.

## Conclusion

We recommend that individual sites considering active MRSA surveillance make decisions about testing methods based upon local conditions, including known risk groups, carriage and infection rates, organizational structures, and hygiene policies. The Hain GenoQuick MRSA is the most advantageous method for MRSA detection in centralized surveillance programs, while GeneXpert SA Nasal complete assay is better suited to high-risk settings in need of rapid results. Where resources are limited, CHROMagar MRSA II culture-based method is a rational option, and whose sensitivity can be improved by swabbing of multiple anatomic sites.

## Data Availability

Except for participant identifying information, all data generated or analysed during this study are included in this published article.

## Abbreviations

ATCC: American Type Culture Collection
BD: Becton Dickinson
CRS: Composite Reference Standard
CT: Cycle threshold
DNA: Deoxyribonucleic acid
HIV: Human Immunodeficiency Virus
ISS: Immune Suppression Syndrome
*mecA*: Methicillin resistance gene
MRRH: Mbarara Regional Referral Hospital
MRSA: Methicillin-resistant *Staphylococcus aureus*
MSSA: Methicillin-Susceptible *Staphylococcus aureus*
PCR: Polymerase Chain Reaction
REDCap: Research Electronic Data Capture
SA: *Staphylococcus aureus*
SCC: Staphylococcal cassette chromosome
*spa*: Staphylococcal protein A gene
